# Resting‐State Functional Magnetic Resonance Imaging Reveals the Effects of rTMS on Neural Activity and Brain Connectivity After Experimental Stroke

**DOI:** 10.1111/cns.70104

**Published:** 2024-11-04

**Authors:** Jian Hu, Yan Hua, Congqin Li, Anjing Zhang, Yuyuan Wang, Yulong Bai

**Affiliations:** ^1^ Department of Rehabilitation Medicine, Huashan Hospital Fudan University Shanghai China; ^2^ Shanghai First Rehabilitation Hospital Shanghai China

**Keywords:** continuous theta burst stimulation, intermittent theta burst stimulation, repetitive transcranial magnetic stimulation, resting‐state functional magnetic resonance imaging, stroke

## Abstract

**Aims:**

Limited understanding of neurobiological mechanisms of repetitive transcranial magnetic stimulation (rTMS) prevents us from choosing optimal therapeutic regimen for patients to improve therapeutic efficiency. Resting‐state functional magnetic resonance imaging (rs‐fMRI) has been demonstrated to obtain comparable functional readouts across species.

**Methods:**

Intermittent and continuous theta burst stimulation were used to stimulate ipsilesional and contralesional hemisphere, respectively, during the subacute phase after stroke. We used a rat middle cerebral artery occlusion stroke model. The amplitude of low‐frequency fluctuations and functional connectivity analyses of rs‐fMRI were chosen to detect neuron activity and functional connectivity. The expression of neuron activation marker c‐Fos and axonal plasticity marker GAP43 was examined by an immunochemistry method to corroborate the results of rs‐fMRI.

**Results:**

iTBS altered the long‐term neuronal activity in bilateral sensorimotor cortex, whereas cTBS influenced immediate neuronal activity of bilateral sensorimotor cortex. In addition, cTBS enhanced interhemispheric and intrahemisheric functional connectivity in contralesional hemisphere, accompanied by axonal and dendritic remodeling in the perilesional cortical areas and contralesional homologous areas after large stroke.

**Conclusion:**

rTMS exerted complex effects on brain structural and functional connectivity in addition to affecting cortical excitability. cTBS promoted the compensatory effect of contralesional hemisphere after stroke with large lesions.

## Graphical abstract

Intermittent and continuous theta burst stimulation were used to stimulate ipsilesional and contralesional hemisphere, respectively, during the subacute phase after stroke. Rs‐fMRI was used to detect neuronal activity and functional connectivity, and immunohistochemistry was used to verify the results of rs‐fMRI.

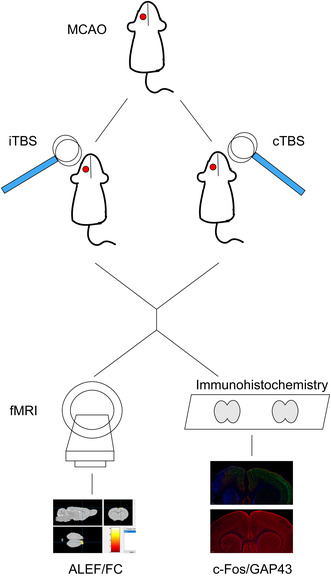

## 1 Introduction

Stroke is the leading cause of long‐term disability globally and carries a significant economic burden [1]. Repetitive transcranial magnetic stimulation (rTMS) has received much attention in recent years because of its ability to effectively improve motor relearning and motor recovery after stroke, especially the motor performance of hemiplegic upper limb and hand [2].

Excitatory information from one hemisphere is transmitted through the corpus callosum (CC) to the contralateral hemisphere, activating inhibitory cells and thereby inhibiting activity [3]. This interhemispheric inhibition is important for the flexibility of bilateral limb movements and is required for the integration of bilateral sensory and motor signals [4]. Stroke leads to the weakening of the inhibitory influence of the affected hemisphere on the unaffected hemisphere, which disrupts the balance across hemispheres [5]. rTMS has been used to promote functional recovery after stroke by affecting this excitation–inhibition balance, such as up‐regulating the cortical excitability of the affected hemisphere or down‐regulating the cortical excitability of the unaffected hemisphere. However, evidence from clinical studies has shown inconsistent treatment responses. Some studies have shown that rTMS promotes motor recovery [6], while other studies have shown negative results [7]. This is attributed to our limited understanding of its neurobiological mechanisms. It also prevents us from choosing optimal therapeutic regimen for patients to improve therapeutic efficiency.

Animal studies provide the possibility to explore the effects of rTMS on brain functional and structural reorganization after stroke. In addition to the research of molecular and cellular processes, previous studies have confirmed that resting‐state functional magnetic resonance imaging (rs‐fMRI) could obtain comparable functional readouts across species [8, 9, 10]. rs‐fMRI detects brain activity in the resting state according to the low‐pass filtering of spontaneous blood oxygenation level‐dependent fMRI signals [11]. The amplitude of low‐frequency fluctuations (ALFF) and functional connectivity (FC) analyses are commonly used rs‐fMRI analyses. ALFF has been proven to be effective and reliable in revealing spontaneous neuronal early metabolic activity [12, 13]. ALFF can directly reflect the intensity of neuron spontaneous activity. Increased ALFF value indicates neuronal activation in the corresponding brain regions. FC analysis reflects the level of FC between different brain areas [14]. Focal cerebral ischemia often leads to widespread disruption of FC, which is associated with neurological impairment and functional recovery. Previous studies have consistently found that reduced interhemispheric connectivity between homotopic cortical areas, such as the sensorimotor network, correlates with the severity of injury and functional recovery, whether in humans or in rodents [8, 9, 15].

In the present study, we used a rat middle cerebral artery occlusion stroke model. Intermittent and continuous theta burst stimulation, which are unique forms of rTMS with stronger efficiency in modulating cortical excitability, were used to stimulate ipsilesional and contralesional hemisphere, respectively, during the subacute phase. ALEF and FC analyses of rs‐fMRI were chosen to detect neuron activity and FC after stimulation. In addition, the expression of neuron activation marker c‐Fos and axonal plasticity marker GAP43 was examined by an immunochemistry method to corroborate the results of rs‐fMRI. The aim of this study was to investigate the effects of iTBS or cTBS on brain structure and function after stroke.

## 2 Material and Methods

### 2.1 Animals

Adult male Sprague–Dawley rats between 8 and 9 weeks of age, weighing 260–280 g, were obtained from Beijing Vital River Laboratory Animal Technology Co. Ltd., Beijing, China (license No. SYXK (Hu) 2020–0032). Rats were housed in the SPF animal housing area of the Department of Laboratory Animal Science, Fudan University, and were provided food and water AD libitum under a 12‐h light/dark cycle. All experimental procedures in this study were in line with ethical standards of the Animal Welfare and Ethics Group, Department of Laboratory Animal Science, Fudan University (2021JS Huashan Hospital‐139).

### 2.2 Middle Cerebral Artery Occlusion (MCAO)

Rats were anesthetized with 5% isoflurane for induction and 1.5% isoflurane combined with 0.05 mg/kg dexmedetomidine for maintenance. Poly‐L‐lysine–coated filaments were inserted into the left middle cerebral artery for 90 min. In order to ensure the middle cerebral artery occlusion, the length of the filament in the internal carotid artery was 18–20 mm. The diameter of poly‐L‐lysine–coated blunted tip was 0.36 ± 0.02 mm, and the diameter of the filament was 0.26 mm. After 90 min of focal ischemia, filaments were released to allow reperfusion. Body temperature was maintained at 37°C through a heating pad. After surgery, rats were returned to their cages and the bedding was cleaned. Rats with mNSS scores of 6–12 were included; the rats were randomly divided into control group, iTBS group, and cTBS group.

### 2.3 Repetitive Transcranial Magnetic Stimulation (rTMS) Treatment

Awake rats were manually immobilized during rTMS treatment. The rTMS intensity was determined according to the twitch threshold of the proximal forelimb of the rat, and the stimulus output was set to 80% of the twitch threshold. The twitch threshold was defined as the minimum intensity to elicite a visible twitch in the proximal forelimb ipsilateral to the affected hemisphere [16]. This is because the twitch reaction in the rat's distal forelimb was not visible. In addition, the intensity required to induce twitch on the paralyzed limb was often too high or even exceeded the maximum output intensity of the machine. The twitch threshold was identified when twitch reactions were evoked at least five in ten consecutive trials.

TMS was performed with a magnetic stimulator (CCY‐I, Wuhan Yiruide Medical Equipment, Wuhan, China) and a circular coil (6 cm in diameter). The center of the coil was 10 mm above the primary motor cortex (M1). iTBS targeted the ipsilaeional M1, whereas cTBS targeted the contralesional M1. The magnetic field intensity at the center of the coil 10 mm away from the scalp was 1.58 T. The twitch threshold was 55%–60% of the maximum output of the stimulator. Theta burst stimulation referred to the classical protocols proposed by Huang et al. [17] One iTBS block consisted of 20 trains, each of which repeated ten 50 Hz bursts (three pulses) at 5 Hz, and then rest for 8 s, for a total of 192 s. One cTBS block was a single 40 s train that repeated 50 Hz bursts (three pulses) at 5 Hz. Both TBS protocols contained 600 pulses. For sham stimulation, the distance from the coil to the brain was increased to 100 mm. Rats in the control group received sham stimulation. The rats were treated with rTMS five times a week for 2 weeks starting on day 4 after stroke, ten sessions in total.

### 2.4 Behavioral Test

Behavior tests were carried out at 4 and 17 days after stroke, including modified neurological severity score (mNSS) and the ladder rung walking task test. The mNSS includes motor, sensory, reflex, and balance tests, with a maximum score of 18 [18]. The ladder rung walking task was performed as our previous study [19]. All rats underwent 3 days of pre‐training prior to stroke. The test was conducted in a quiet room, and the rats crossed the ladder three times for each test. The test was recorded with a camera, and each step of the paralyzed forelimb was scored according to the video. The scoring criteria were as follows: (0) total miss, [1] deep slip, [2] slight slip, [3] replacement, [4] correction, [5] partial placement, and [6] correct placement. The average score of the paralyzed forelimb was calculated for statistical analysis.

### 2.5 Golgi‐Cox Staining

Golgi‐cox staining was used to visualize the dendritic branches. Rats were anesthetized and sacrificed by decapitation at 17 days after MCAO. We used the FD Rapid Golgi staining kit (FD Neuro Technologies), and the staining protocol was referred to our previous study [19]. Target neurons that were clearly visible and had few crossing with other neurons were selected and photographed under a 20x magnification objective. Ten neurons were selected from layer 4 and 5 cortex in each brain region. The basal dendrites of neurons were manually delineated using the NeuronJ plugin of Image J software (National Institutes of Health, Bethesda, MD, USA), and the number and sum length of the dendritic branches were calculated.

### 2.6 MRI Acquisition

All MRI was acquired using a 11.7 T animal scanner (Bruker Corporation, Germany). FMRI scans were performed at 7 days after rTMS treatment (*n* = 5 per group). All the rats were anesthetized with 5% isoflurane for induction and 1.5% isoflurane combined with 0.05 mg/ kg dexmedetomidine for maintenance. The rs‐fMRI scans were acquired with a spin‐echo echo‐planer (SE‐EPI) sequence: repetition time (TR) = 2000 ms, echo time (TE) = 12.8 ms, the field of view (FOV) =30 × 30 mm, and slice thickness = 0.5 mm. The anatomical images (T2 images) were acquired by a spin‐echo (Turbo‐RARE) sequence. The T2 image sequence parameters were TR = 5000 ms, TE = 25 ms, FOV = 30 × 30 mm, and slice thickness = 0.5 mm.

### 2.7 MRI Data Analysis

The statistical Parametric Mapping 12 (SPM12, http://www.fil.ion.ucl.ac.uk/spm/), resting‐state fMRI Data Analysis Toolkit (REST, http://www.restfmri.net/forum), and DPABI (http://rfmri.org/dpabi) on the MATLAB platform were used to process the rs‐fMRI data and calculate the indicators. The data processing steps were as follows [20, 21]: (1) format conversion, (2) the removal of first ten time points, (3) voxel augment, (4) slice timing, (5) realign, (6) reorientation, (7) normalization, (8) smooth, (9) removal of the linear trend, and (10) indicator calculation: ALFF, mean ALEF (mALEF), and fractional ALEF (fALEF) values were calculated using DPABI and band filtered (0.01–0.08 Hz) to reduce noise. ALFF values for each voxel were normalized by z‐score transformation. The mALEF, zALEF, mfALEF, and zfALEF values were statistically analyzed. Regions of interest (ROIs) were extracted using REST based on SIGMA anatomical brain atlas. In the present study, 18 ROIs, including the left and right primary motor cortex (M1), sencondary motor cortex (M2), primary somatosensory cortex (S1), secondary somatosensory cortex (S2), primary somatosensory cortex, forelimb region (S1FL), primary somatosensory cortex, hindlimb region (S1HL), Cg1 (cingulate cortex, area 1), CC, and STR (striatum) were used to perform seed‐based FC analysis. Two‐sample t test was used for comparison between each two groups (control group vs. iTBS group; control group vs. cTBS group). A single voxel threshold of *p* < 0.001 combined with cluster‐level false discovery rate (FDR) correction was regarded as significant. The statistically significant clusters were mapped to the rat SIGMA atlas for presentation via xjView (https://www.alivelearn.net/xjview/). Brain regions with clusters larger than ten voxels were reported.

### 2.8 Immunohistochemistry

Rats were anesthetized with 5% isoflurane and transcardially perfused with saline and 4% paraformaldehyde. After gradient dehydration in sucrose solution, brains were frozen and cut into 20 μm thickness sections on a freezing microtome. The sections were blocked for 1 h with 10% goat serum at room temperature and then incubated with the primary antibodies at 4°C overnight. The following primary antibodies were used: anti‐c‐Fos (GB11069‐100, Servicebio) and anti‐GAP43 (GB11095‐100, Servicebio). Sections were then washed and incubated with appropriate secondary antibodies for 1 h at room temperature. To label nuclei, the sections were stained with 4’,6―diamidino‐2―phenylindole (DAPI) for 10 min at room temperature. The slices were scanned with Pannoramic scanner (3DHISTECH, Hungary) and photographed with CaseViewer 2.4 (3DHISTECH, Hungary). The images were processed and analyzed using ImageJ software.

### 2.9 Statistical Analysis

Data analysis was carried out by Prism v10 software (GraphPad, La Jolla, CA, USA). All data were tested for normality by the Kolmogorov–Smirnov test. If the variances were homogeneous, two‐sample t test was used for comparison between each two groups (control group vs. iTBS group; control group vs. cTBS group). If the variances were not homogeneous, the Mann–Whitney test was used. The mean value was reported together with the standard deviation (SD). Differences between groups were judged as significant if the *p* value was < 0.05.

## 3 Results

### 3.1 iTBS and cTBS Promoted Motor Function Recovery in Cerebral Ischemia Rats

**FIGURE 1:**
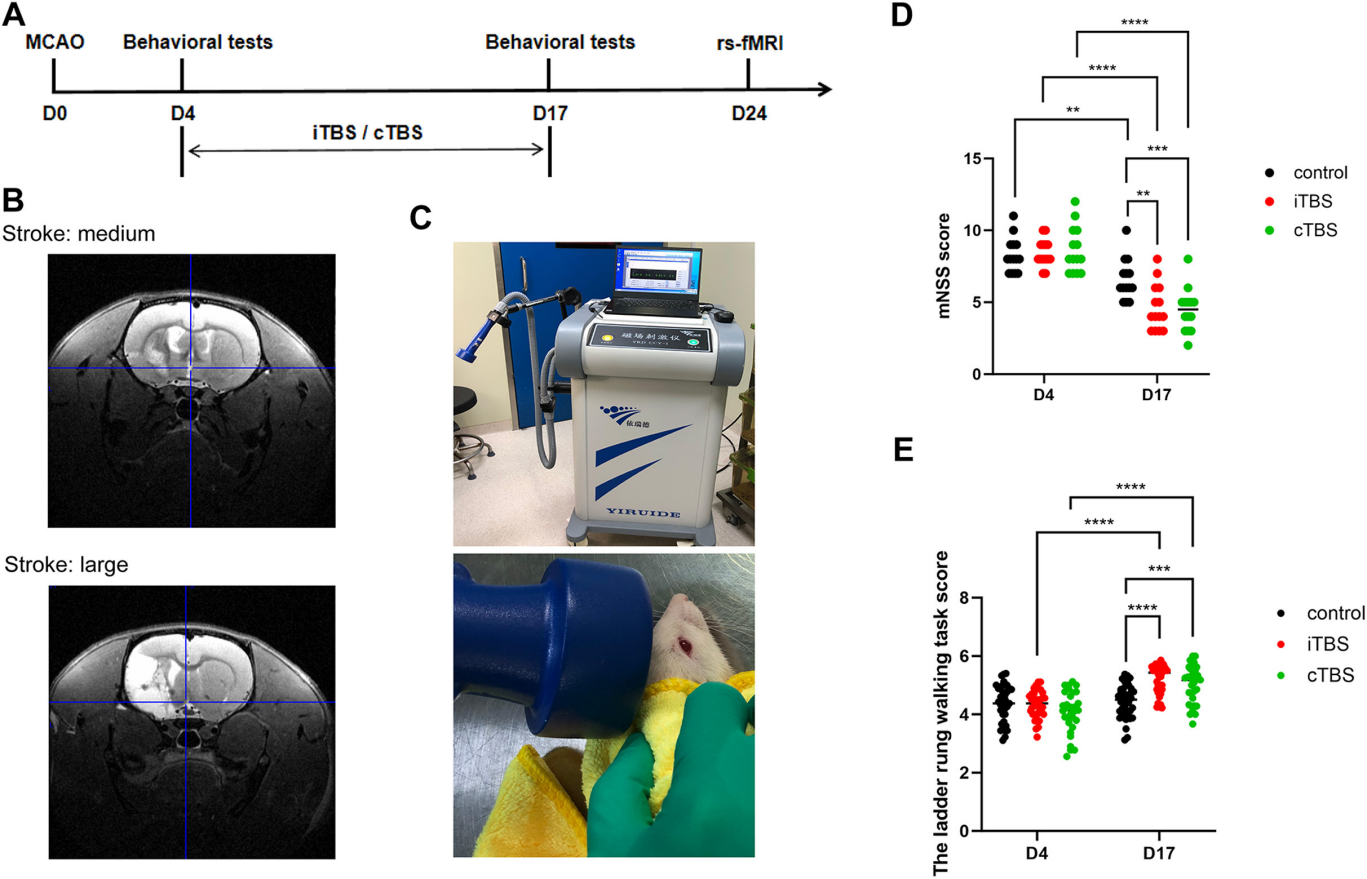
iTBS and cTBS improved motor function of rats after stroke. (A) Experimental design of the study. (B) T2 anotomical images denmonstrated left‐sided ischemic lesions. Medium stroke (stroke: Medium) was characterized by mainly subcortical damage with occasional involvement of some ventrolateral cortical tissue. Large stroke (stroke: Large) had extensive damage in subcortical and ventrolateral as well as dorsolateral cortical tissue. (C) Schematic representation of rTMS treatment. (D, E) The baseline and post‐intervention scores in the mNSS score and ladder rung walking task test were compared. (*n* = 14, per group. *p < 0.05, **p < 0.01, ***p < 0.001, ****p < 0.0001).

The mNSS and the ladder rung walking task test were used to evaluate the motor function of rats. These results suggested that both iTBS and cTBS improved motor function of cerebral ischemia rats (Figure 1D,E).

### 3.2 ALEF Values Were Altered After iTBS Intervention but Not After cTBS Intervention

**FIGURE 2:**
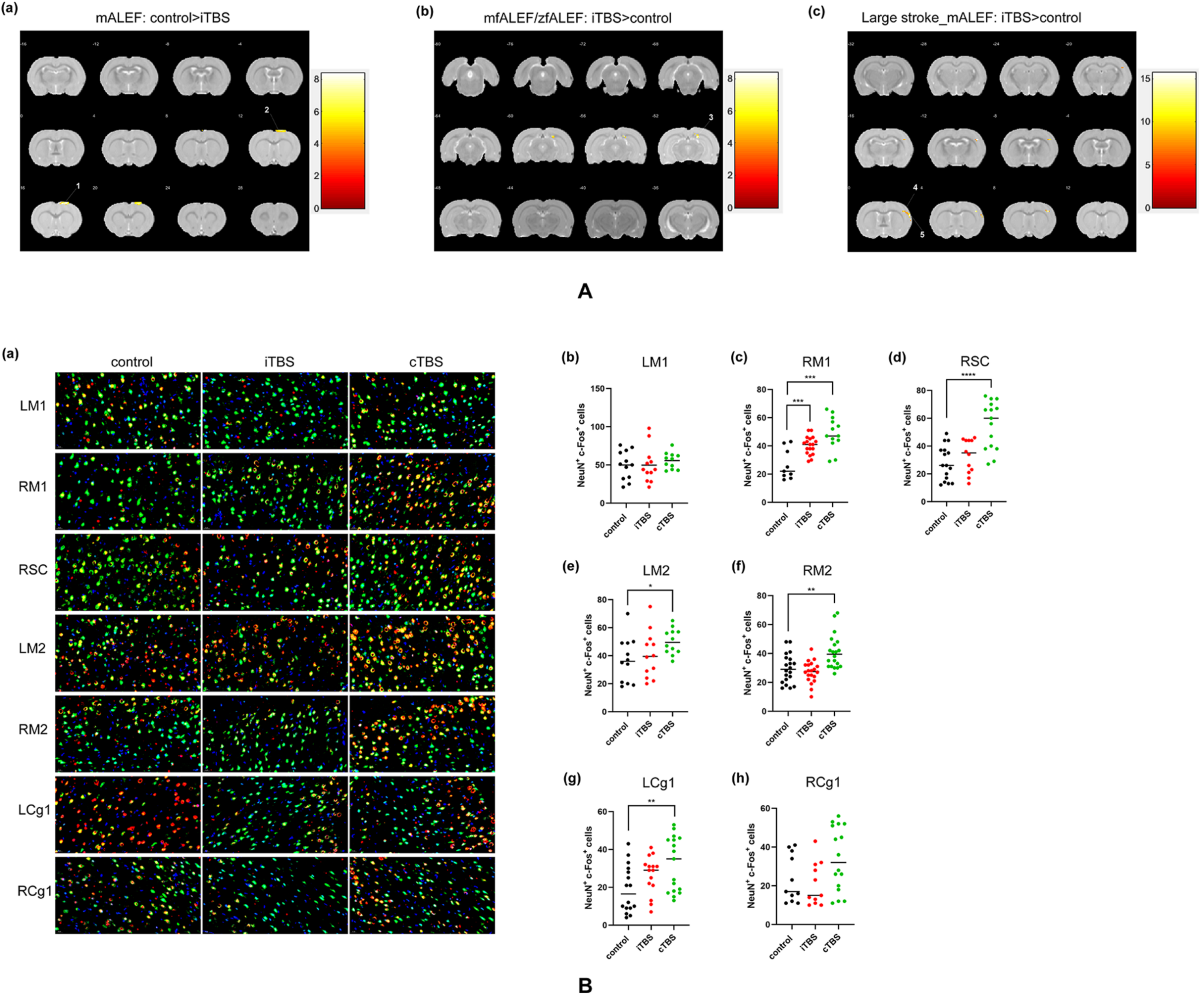
Effect of iTBS and cTBS on the immediate and long‐term neural activity. (A) Brain maps for ALFF. The left side of the image corresponds to the left side of the brain in the coronal plane. The colorbar is to the right of each map, and the map shows positive values. (a) mALEF, control > iTBS. The significant level was set at *p* < 0.001 and cluster size > 65 voxels, FDR corrected. (b) mfALEF/zfALEF, iTBS > control. The significant level was set at *p* < 0.001 and cluster size > 22 voxels, FDR corrected. (c) Large stroke, mALEF, iTBS > control. The significant level was set at *p* < 0.001 and cluster size > 15 voxels, FDR corrected. (1, right primary motor cortex; 2, right secondary motor cortex; 3, right corpus callosum and associated subcortical white matter; 4, right primary somatosensory cortex barrel field; and 5, right primary somatosensory cortex, upperlips.) (B) c‐Fos activation. (a) Representative images of double‐labeled immunofluorescence staining of c‐Fos (red) and NeuN (green) in different brain areas. Scale bars: 20 μm. (b–h) Comparison of c‐Fos‐positive cells between groups in different brain regions. LM1(b), RM1(c), RSC(d), LM2(e), RM2(f), LCg1(g), and RCg1(h). (*n* = 5, per group. LM1, left primary motor cortex; RM1, right primary motor cortex; LM2, left secondary motor cortex; RM2, right secondary motor cortex; RSC, right sensory cortex; LCg1, left cingulate cortex, area 1; RCg1, right cingulate cortex, area 1. *p < 0.05, **p < 0.01, ***p < 0.001, ****p < 0.0001.)

In the present study, mALEF, zALEF, mfALEF, and zfALEF were all calculated to quantify the neural activity. Compared with the control group, the iTBS group showed significantly decreased mALEF values in the right M1 and M2 and significantly increased mfALEF/zfALEF values in the right CC (Table 1) (Figure 2A,a,b). The results indicated that iTBS corrected the abnormal hyperexcitability state of contralesional motor cortex after stroke, and it may be transmitted through the CC. When the comparison was conducted only in rats with large (cortical and subcortical) lesions, we found that iTBS significantly increased mALEF values in the right primary somatosensory cortex, upperlips area, and barrel field area compared with the control group (Table 1) (Figure 2A,c). This suggested that iTBS may enhance the compensation in the contralesional sensory cortex after large cerebral infarction. There were no significant differences between the cTBS group and the control group, either in all rats or in the large stroke subgroup.

**TABLE 1:** Brain regions showed significant inter‐group differences of ALFF values.

Contrast		Cluster	Sub‐regions	Voxel size	Peak t‐value	Peak MNI Coordinates (mm)		
						x	y	z
Control > iTBS	mALEF	Cluster 1		65	8.36	29	15.95	63.2
			Primary motor cortex R	31	7.71	26	15.95	60.2
			Secondary motor cortex R	14	7.94	17	15.95	60.2
iTBS > Control	mfALEF	Cluster 1		22	8.06	23	−56.05	48.2
			Corpus callosum and associated subcortical white matter R	19	8.06	23	−56.05	48.2
	zfALEF	Cluster 1		27	8.15	23	−53.05	45.2
			Corpus callosum and associated subcortical white matter R	22	8.15	23	−53.05	45.2
Large stroke								
iTBS > Control	mALEF	Cluster 1		31	15.69	50	6.95	39.2
			Primary somatosensory cortex upperlips R	17	15.14	68	0.95	27.2
		Cluster 2		15	15.44	56	−11.05	39.2
			Primary somatosensory cortex barrel field R	15	15.44	56	−11.05	39.2

### 3.3 rTMS‐Induced c‐Fos Activation in the Sensorimotor Cortex and Cingulate Cortex

c‐Fos is a widely recognized immediate‐early gene marker of neuronal activity, and its expression peaks within 1–3 h after stimuli [22]. The rats were sacrificed within 2 h after the last rTMS treatment to measure the expression of c‐Fos. According to the results of ALEF, the target brain regions were selected, including bilateral M1, M2, Cg1, and right sensory cortex. The left sensory cortex was excluded because of extensive tissue defects due to ischemic infarction. Compared with the control group, the cTBS group showed significantly increased number of c‐Fos‐positive neurons in the bilateral M2, left Cg1, right M1, and right sensory cortex (Figure 2B,c–g). However, only the number of c‐Fos‐positive neurons in the right M1 was significantly higher in the iTBS group than in the control group (Figure 2B,c). These results suggested that cTBS could immediately increase neuron activity in the perilesional areas and contralesional homologous areas.

### 3.4 cTBS Enhanced Functional Connectivity in the Contralesional Hemisphere After Large Stroke

**FIGURE 3:**
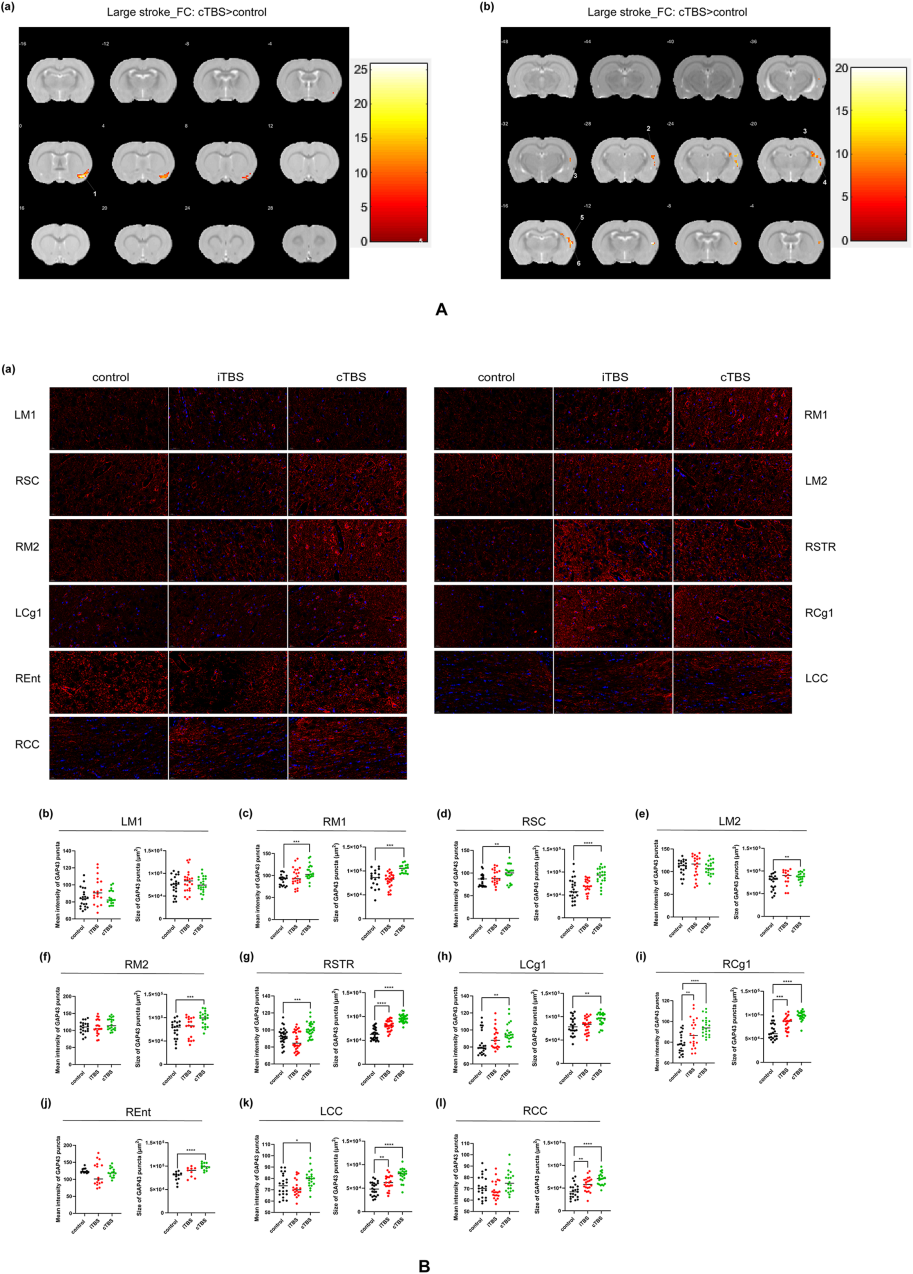
Effect of iTBS and cTBS on the functional connectivity and axonal remodeling in bilateral hemisphere. (A) Brain maps for functional connectivity. The left side of the image corresponds to the left side of the brain in the coronal plane. The colorbar is to the right of each map, and the map shows positive values. (a) Seed regions for FC analysis were bilateral M1, M2, Cg1, right S1FL, and S1HL, and these seed regions all showed enhanced functional connectivity with right entorhinal cortex. The significance level was set at *p* < 0.001 and cluster size > 74 voxels, FDR corrected. (b) Seed region for FC analysis was right STR. The significance level was set at *p* < 0.001 and cluster size > 99 voxels, FDR corrected. (M1, primary motor cortex; M2, secondary motor cortex; Cg1, cingulate cortex, area 1; S1FL, primary somatosensory cortex, forelimb region; S1HL, primary somatosensory cortex, hindlimb region; STR, striatum; 1, right entorhinal cortex; 2, right secondary auditory cortex dorsal part; 3, right corpus callosum and associated subcortical white matter; 4, right primary somatosensory cortex barrel field; 5, right primary somatosensory cortex, upperlips; and 6, right secondary somatosensory cortex.) (B) GAP43 immunofluorescence staining. (a) Representative images of immunofluorescence staining of GAP43 in different brain areas. Scale bars: 20 μm. (b–l) Comparison of GAP43 expression between groups in different brain regions. LM1(b), RM1(c), RSC(d), LM2(e), RM2(f), RSTR(g), LCg1(h), RCg1(i), REnt(j), LCC(k), and RCC(l). (*n* = 5, per group. LM1, left primary motor cortex; RM1, right primary motor cortex; LM2, left secondary motor cortex; RSC, right sensory cortex; RM2, right secondary motor cortex; RSTR, right striatum; LCg1, left cingulate cortex, area 1; RCg1, right cingulate cortex, area 1; REnt, right entorhinal cortex; LCC, left corpus callosum; RCC, right corpus callosum. *p < 0.05, **p < 0.01, ***p < 0.001, ****p < 0.0001.)

Unfortunately, in the comparison of all rats without discrimination of lesion size, FC based on 18 seed regions (bilateral M1, M2, S1, S2, S1FL, S1HL, Cg1, CC, and STR) did not show significant difference between groups. Here, the FC data from rats with large lesions was also analyzed (*n* = 3 for each group). When the seeds were located in the bilateral M1, bilateral M2, right S1FL and S1HL, and bilateral Cg1 and right CA1, the cTBS group showed significantly increased FC in the right Ent compared with the control group (Figure 3A,a). When the seed was located in the right STR, the cTBS group showed significantly increased FC in several regions, involving the right primary somatosensory cortex, barrel field area and upperlips area, right S2, right secondary auditory cortex dorsal part, and right CC (Figure 3A,b). These results suggested that cTBS could enhance the FC in the contralesional hemisphere after large stroke, in which the entorhinal cortex and striatum played an important role (Table 2).

**TABLE 2:** cTBS increased strengths of functional connectivity after stroke with large lesion.

Connected region	Contrast	Cluster	Sub‐regions	Voxel size	Peak t‐value	Peak MNI coordinates (mm)		
						x	y	z
LM1	cTBS > Control	Cluster 1		74	25.80	56	0.95	−14.8
			Entorhinal cortex R	63	25.80	56	0.95	−14.8
LM2	cTBS > Control	Cluster 1		74	27.04	56	0.95	−14.8
			Entorhinal cortex R	63	27.04	56	0.95	−14.8
RM1	cTBS > Control	Cluster 1		74	25.90	56	0.95	−14.8
			Entorhinal cortex R	63	25.90	56	0.95	−14.8
RM2	cTBS > Control	Cluster 1		74	25.57	56	0.95	−14.8
			Entorhinal cortex R	63	25.57	56	0.95	−14.8
RS1FL	cTBS > Control	Cluster 1		74	26.46	56	0.95	−14.8
			Entorhinal cortex R	63	26.46	56	0.95	−14.8
RS1HL	cTBS > Control	Cluster 1		73	26.22	56	0.95	−14.8
			Entorhinal cortex R	62	26.22	56	0.95	−14.8
LCg1	cTBS > Control	Cluster 1		74	27.13	56	0.95	−14.8
			Entorhinal cortex R	63	27.13	56	0.95	−14.8
RCg1	cTBS > Control	Cluster 1		74	25.28	56	0.95	−14.8
			Entorhinal cortex R	63	25.28	56	0.95	−14.8
RSTR	cTBS > Control	Cluster 1		99	19.91	62	−11.05	15.2
			Primary somatosensory cortex barrel field R	21	15.52	68	−23.05	30.2
			Secondary somatosensory cortex R	21	19.91	62	−11.05	15.2
			Primary somatosensory cortex upperlips R	17	11.42	65	−26.05	24.2
			Secondary auditory cortex dorsal part R	15	16.71	68	−26.05	9.2
			Corpus callosum and associated subcortical white matter R	10	14.06	35	−8.05	30.2

Abbreviations: LCg1, left cingulate cortex, area 1; LM1, left primary motor cortex; LM2, left secondary motor cortex; RCg1, right cingulate cortex, area 1; RM1, right primary motor cortex; RM2, right secondary motor cortex; RS1FL, right primary somatosensory cortex, forelimb region; RS1HL, right primary somatosensory cortex, hindlimb region; RSTR, right striatum.

### 3.5 Effects of iTBS and cTBS on the Axonal Remodeling in Seed Regions

Similarly, target brain regions were selected based on rs‐fMRI results to detect the expression of axonal marker GAP43 in rats with large stroke, including bilateral M1, M2, Cg1, CC and right sensory cortex, Ent, and STR. Compared with the control group, the cTBS group showed significantly increased expression of GAP43 in the right M1, sensory cortex, STR and Ent, bilateral M2, Cg1, and CC (Figure 3B,c–l). The expression of GAP43 in the right Cg1 and STR, bilateral CC was significantly increased in the iTBS group compared with the control group (Figure 3B,g,i,k,l). These results indicate that cTBS enhanced axon remodeling in a wide range of brain regions. However, iTBS showed no significant effect on the axon remodeling of sensorimotor cortex.

### 3.6 Effects of iTBS and cTBS on the Dendritic Plasiticity in Seed Regions

**FIGURE 4:**
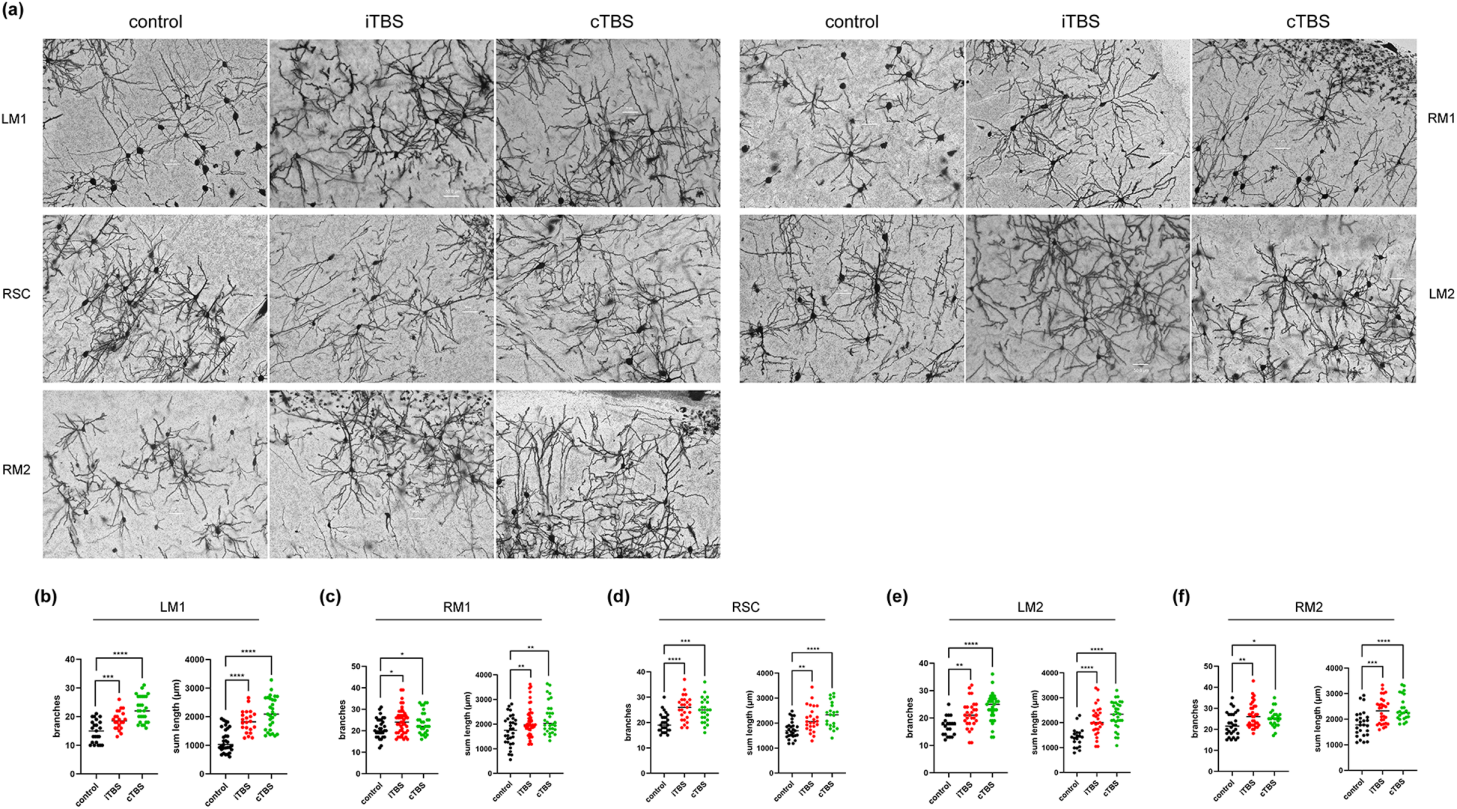
Effect of iTBS and cTBS on the dendritic plasticity in bilateral hemisphere. (a) Representative image of Glogi straining in different brain areas. Scale bars: 50 μm. (b–f) Comparison of dendritic branches and length between groups in different brain regions. LM1(b), RM1(c), RSC(d), LM2(e), and RM2(f). (*n* = 4, per group. LM1, left primary motor cortex; RM1, right primary motor cortex; RSC, right sensory cortex; LM2, left secondary motor cortex; RM2, right secondary motor cortex. *p < 0.05, **p < 0.01, ***p < 0.001, ****p < 0.0001.)

Unlike the results for GAP43, we found that both iTBS and cTBS promoted dendrite remodeling in the bilateral M1 and M2 and right sensory cortex compared with the control group (Figure 4). These results indicated that both iTBS and cTBS enhanced the dendritic plasiticity in bilateral sensorimotor cortex.

## 4 Discussion

In the present study, we used excitatory stimulation iTBS to target the affected hemisphere and inhibitory stimulation cTBS to target the unaffected hemisphere, which was consistent with the routine clinical application protocol. Interestingly, the results of rs‐fMRI suggested that iTBS altered the neuronal activity in the sensorimotor cortex after stroke, whereas cTBS enhanced interhemispheric and intrahemispheric FC only after large stroke. This was supported by the expression of neuronal activity marker c‐Fos, axon marker GAP43, and dendritic plasticity. We did not find significant differences between the iTBS and cTBS groups in the behavioral tests. Therefore, we did not discuss in detail any other outcomes between these two groups (the data not shown).

### 4.1 iTBS and cTBS Differentially Altered the Neuronal Activity in the Bilateral Sensorimotor Cortex and Cingulate Cortex

The results of ALEF suggested that iTBS decreased the neuronal activity in the contralesional M1 and M2 and increased the neuronal activity in the contralesional CC. However, c‐Fos positive neurons in the contralesional M1 increased significantly after iTBS intervention. This inconsistency may be due to the following two reasons: first, c‐Fos reflects the immediate neuronal response after TMS treatment, whereas in this study, rs‐fMRI was performed at 7 days after rTMS treatment. Zhong et al. found that the activation of primary sensory cortex evoked by 10 Hz rTMS was evident 30 min after stimulation but disappeared 3 days after stimulation [23]. Thus, rs‐fMRI reflected the long‐term neuronal activity in the resting state after rTMS treatment in this study. Second, it is possible that the increased c‐Fos‐positive neurons in the contralesional M1 were inhibitory neurons that are activated by excitatory stimulation from the ipsilesional M1 after iTBS treatment. Unfortunately, neuron types were not distinguished in this study. In addition, we found that iTBS increased ALEF values in the contralesional sensory cortex in rats with large lesion. Most of the tissue in the ipsilesional sensory cortex was lost after cerebral ischemia with large lesion. Therefore, we suggested that the contralesional sensory cortex plays a compensatory role in the large lesion. In contrast to us, chronic optogenetic excitation of contralesional primary somatosensory forepaw cortex inhibited behavioral recovery and cortical remapping after photothrombosis of ipsilesional primary somatosensory forepaw cortex [24]. This is because the damage caused by photothrombotic stroke is mild, and remodeling of peri‐infarct cortex was associated with behavioral recovery. However, many studies have suggested that the compensatory effect of contralesional sensorimotor cortex is essential for the recovery of sensory motor function after moderate to severe stroke [25, 26, 27].

Although cTBS had no significant effect on ALEF values, the results of c‐Fos staining suggested that cTBS increased immediate neuronal activity in a wide range of regions, including bilateral M2, left Cg1, right M1, and right sensory cortex. This indicated that cTBS promoted functional reorganization in the perilesional areas and homologous cortical areas in the contralesional hemisphere.

### 4.2 cTBS Enhanced Structural and Functional Connectivity in the Perilesional Cortical Areas and Contralesional Hemisphere in Rats After Stroke With Large Lesion

In the FC analysis, we found no significant difference between groups in the comparison of all rats without discrimination of lesion size. It may be due to the inconsistency in lesion area, and our sample size was small. Thus, the data of rats with large lesion were analyzed again. Interestingly, we found that cTBS enhanced the FC between contralesional entorhinal cortex with bilateral M1, M2, Cg1, and contralesional sensory cortex. Entorhinal cortex is the peri‐hippocampal cortex that innervates hippocampal regions, and the hippocampus projects directly back to the entorhinal cortex [28]. Entorhinal cortex is involved in memory function and entorhinal cortex‐deep brain stimulation has shown a promising effect on enhancing memory both in patient and animal studies [29, 30, 31, 32]. Previous studies have demonstrated that the hippocampal‐cortex resting‐state FC was associated with memory impairment [33, 34]. The injury of hippocampal and cortical regions after stroke caused information transfer obstacle, leading to impaired memory [35]. A recent study suggested that enriched environment intervention increased interhemispheric connectivity between the hippocampus and entorhinal cortex and ameliorated post‐stroke memory deficits [21]. In this study, we did not find altered FC between the hippocampus and entorhinal cortex after treatment (data not shown). However, cTBS increased interhemispheric and intrahemisheric FC between contralesional entorhinal cortex and sensorimotor cortex and cingulate cortex. The recovery of motor function after stroke is a process of motor relearning. Therefore, we suggested that cTBS promoted motor function recovery by affecting learning and memory. Moreover, it is possible that the contralesional entorhinal cortex played an important compensatory role when lesions invloved in ipsilesional hippocampus as suggested in the study by Lu et al. [21] In addition, cTBS increased intrahemisheric FC between striatum and sensory cortex and CC in the contralesional hemisphere. In brief, these results strongly demonstrated that cTBS promoted the compensatory effect of contralesional hemisphere that was beneficial to motor function recovery after moderate to severe stroke. Consistent with FC, cTBS enhanced the axon remodeling in the perilesional area and contralesional homologous areas. van Meer et al. also demonstrated the increased intrahemispheric FC in the contralesional sensorimotor cortex and the strong link between functional and structural reorganization of neuronal networks after a large unilateral stroke [8, 25].

### 4.3 Strengths and Limitations

We are the first to perform an animal study using iTBS and cTBS to stimulate the affected versus the unaffected cortex after cerebral ischemia, respectively. Furthermore, the rats were stratified into medium stroke and large stroke according to the ischemic lesion. However, there are several limitations in this study. First, the small sample size was insufficient for categorical statistics based on lesion area. Second, we did not specifically label the neuron type and therefore cannot accurately explain the discrepancy between c‐fos activation and ALEF values. Further studies will be based on the results of this study and address these shortcomings.

## 5 Conclusions

In summary, the results of this study are summarized as follows: (1) iTBS altered the long‐term neuronal activity after treatment, while cTBS influenced immediate neuronal activity in a wide range of brain regions involving the perilesional cortical areas and contralesional homologous areas. (2) In the large stroke, cTBS enhanced interhemispheric and intrahemisheric FC in contralesional hemisphere, accompanied by axonal and dendritic remodeling in the perilesional cortical areas and contralesional homologous areas. (3) The results of resting‐state FC, axon remodeling, and dendritic plasticity all supported the superiority of cTBS in promoting the compensatory effect of contralesional hemisphere after stroke with large lesions. Thus, our study suggested that rTMS exerts complex effects on brain structural and FC and dendritic and synaptic plasticity in addition to affecting cortical excitability. iTBS had an advantage in altering cortical excitability, while cTBS had an advantage in altering FC in the whole brain. iTBS primarily affected the sensorimotor cortex, while cTBS affected the brain more broadly. More studies are needed to provide sufficient and robust evidence in the future.

## Author Contributions

**Jian Hu:** conceptualization, funding acquisition, investigation, writing – original draft. **Yan Hua:** methodology, investigation. **Congqin Li:** formal analysis. **Anjing Zhang:** methodology. **Yuyuan Wang:** project administration and funding acquisition. **Yulong Bai:** conceptualization and supervision.

## Conflicts of Interest

The authors declare no conflicts of interest.

## Data Availability

The data that support the findings of this study are available on request from the corresponding author.
